# Characterization of Residential Pesticide Use and Chemical Formulations through Self-Report and Household Inventory: The Northern California Childhood Leukemia Study

**DOI:** 10.1289/ehp.1204926

**Published:** 2012-10-24

**Authors:** Neela Guha, Mary H. Ward, Robert Gunier, Joanne S. Colt, C. Suzanne Lea, Patricia A. Buffler, Catherine Metayer

**Affiliations:** 1 School of Public Health, University of California at Berkeley, Berkeley, California, USA; 2 International Agency for Research on Cancer, Lyon, France; 3 National Cancer Institute, Bethesda, Maryland, USA; 4 Department of Public Health, Brody School of Medicine, East Carolina University, Greenville, North Carolina, USA

**Keywords:** exposure assessment, pesticides, population-based study, residential pesticide use, U.S. EPA

## Abstract

Background: Home and garden pesticide use has been linked to cancer and other health outcomes in numerous epidemiological studies. Exposure has generally been self-reported, so the assessment is potentially limited by recall bias and lack of information on specific chemicals.

Objectives: As part of an integrated assessment of residential pesticide exposure, we identified active ingredients and described patterns of storage and use.

Methods: During a home interview of 500 residentially stable households enrolled in the Northern California Childhood Leukemia Study during 2001–2006, trained interviewers inventoried residential pesticide products and queried participants about their storage and use. U.S. Environmental Protection Agency registration numbers, recorded from pesticide product labels, and pesticide chemical codes were matched to public databases to obtain information on active ingredients and chemical class. Poisson regression was used to identify independent predictors of pesticide storage. Analyses were restricted to 259 participating control households.

Results: Ninety-five percent (246 of 259) of the control households stored at least one pesticide product (median, 4). Indicators of higher sociodemographic status predicted more products in storage. We identified the most common characteristics: storage areas (garage, 40%; kitchen, 20%), pests treated (ants, 33%; weeds, 20%), pesticide types (insecticides, 46%; herbicides, 24%), chemical classes (pyrethroids, 77%; botanicals, 50%), active ingredients (pyrethrins, 43%) and synergists (piperonyl butoxide, 42%). Products could contain multiple active ingredients.

Conclusions: Our data on specific active ingredients and patterns of storage and use will inform future etiologic analyses of residential pesticide exposures from self-reported data, particularly among households with young children.

Numerous epidemiological studies have investigated exposure to pesticides because of concerns about a wide range of health outcomes, including cancer (Steer and Grey 2006). Much of the pesticide exposure in the general population occurs through the use of products in and around the home (Bradman and Whyatt 2005; Grossman 1995; Nigg et al. 1990; World Health Organization 1997) and pesticide residues brought into the home on shoes or clothing from the outdoors or the workplace (Coronado et al. 2006). Pesticide exposure may be greater in certain populations. Living in low-income, urban neighborhoods with poor housing conditions increases the chances of pest infestation and subsequent pesticide use [Department of Housing and Urban Development (HUD) 2006]. Children may experience greater exposure and susceptibility to pesticides because they spend more time at home than adults, exhibit certain behaviors (e.g., hand to mouth, playing on surfaces where pesticide residues may accumulate), have immature metabolism, and are smaller in size (leading to higher consumption of pesticide residues from foods relative to body size) (Steer and Grey 2006).

Assessment of non-occupational pesticide exposure is challenging, despite having benefited from the experience of assessment of occupational exposure to pesticides (Fenske et al. 2005; Zahm et al. 1997), because the general population is typically less able to report histories of use of individual pesticides than farmers and other occupational groups (Zahm and Ward 1998; Zahm et al. 1997). This is further complicated by having to account for the chemical properties of the active ingredient, application method, location of use, handling and knowledge of product toxicity of the person applying the product, and the presence of synergists in the product that could affect dermal uptake (Colt et al. 2007).

Case–control studies assessing non-occupational pesticide exposure have relied largely on self-report of pest treatments via questionnaire, which is limited by potential recall error and a lack of information on the specific active ingredients (Daniels et al. 1997; Infante-Rivard and Weichenthal 2007; Zahm and Ward 1998). Various methods have been employed to improve recall of residential pesticide use (Teitelbaum 2002) such as queries about specific pests treated (in general and also home by home) and lifetime use of pest treatments along a timeline of a participant’s major life events (to establish temporal associations with pesticide exposure) (Fryzek et al. 1997). Self-reported data may be complemented by obtaining dust samples (Colt et al. 2005, 2006; Hartge et al. 2005) or through the use of home inventories to collect information on the presence of specific active ingredients of stored pesticide products (Adgate et al. 2000; Bass et al. 2001; Bradman et al. 1997; Whitmore et al. 1994). However, to date, data are limited regarding active ingredients and patterns of storage and use of residential pesticides. There are few surveys of home and garden pesticides, all published more than a decade ago, in which the pests treated and the active ingredients used are identified (Adgate et al. 2000; Bass et al. 2001; Bradman et al. 1997; Whitmore et al. 1994). An active ingredient is defined as a chemical that prevents, destroys, repels, or mitigates a pest while “inert” or “other” ingredients are all other substances intentionally included in a pesticide product [U.S. Environmental Protection Agency (EPA) 2011].

As part of an integrated assessment of pesticide exposure within a case–control study of childhood leukemia in California, trained interviewers inventoried pesticide products in participants’ homes, queried participants about the pests treated with these products and other products that were not captured during the inventory, and collected dust samples to analyze for the presence of specific active ingredients (Metayer and Buffler 2008). Here we present results from the pesticide inventory in control households with the objectives to describe the patterns of residential pesticide storage and use and identify active ingredients.

## Materials and Methods

*Study population.* Details of the design of the Northern California Childhood Leukemia Study (NCCLS), a population-based case–control study, have been described previously (Bartley et al. 2010; Ward et al. 2009). Briefly, starting in 1995 children newly diagnosed with leukemia were ascertained from pediatric hospitals located in 35 California counties (17 counties in the San Francisco Bay Area and 18 in northern and central California, including the agricultural Central Valley). More than 38% of the births in California between 1995 and 2004 occurred within the 35-county study area (State of California, Department of Finance, Demographic Research Unit 2012). Controls were selected from the California birth registry and individually matched to the cases by child’s date of birth, sex, Hispanic ethnicity, and mother’s race (Bartley et al. 2010; Ma et al. 2004; Ward et al. 2009). A total of 997 leukemia cases and 1,226 controls participated in the NCCLS from 1995 to 2008, including a large proportion of Hispanics (approximately 45%). Eighty-six percent of eligible cases and controls participated in the NCCLS.

From October 2001 to December 2006, a subset of households with children < 8 years old (at diagnosis date for cases or corresponding reference date for controls) who had resided in the same house since the diagnosis/reference date were eligible to participate in a follow-up home visit during which a physical inventory of residential pesticides was conducted (Ward et al. 2009). Of 549 eligible households (244 cases, 305 controls), 500 (241 cases, 259 controls) participated in the inventory study, and 475 households (229 cases, 246 controls) stored at least one pesticide product at the time of interview. Because case households may have changed their habits of pesticide use and storage after the leukemia diagnosis, we restricted this analysis to control households as a better representation of the source population.

The NCCLS was approved by the University of California Committee for the Protection of Human Subjects and the institutional review boards of the National Cancer Institute and all participating hospitals. Written, informed consent was obtained from the parents of all participating children.

*Data collection and record linkage to publicly available databases.* Experienced interviewers were trained to obtain informed consent, administer the interview in English or Spanish, and to use visual aids to obtain information on calendar periods for various time windows of exposure and on location of pesticide use and application methods. Respondents (mainly the mother) directed the interviewer to locations in and around the home, including outdoor sheds and garages, where pesticide products were stored. Interviewers used a standard form, based on a household inventory conducted by the U.S. EPA (Whitmore et al. 1992), to record the name and U.S. EPA registration number appearing on the label for each product, as well as the storage location. Standardized questions were asked about how the product was applied (e.g., ready-to-use spray, flea/tick collar); the purpose of product use (e.g., treatment of ants or cockroaches, fleas or ticks); when the product was last used (e.g., within the past week); the frequency of product use in the previous 12 months (≥ 5 times, < 5 times, never used, don’t know); where the product was used in the previous 12 months (e.g., kitchen); who applied the product in the previous 12 months (biological mother, biological father, child, other); and the specific time periods of use between 3 months before the child’s birth, during the pregnancy, and until his or her third birthday. Information was not collected on the number of households with a garage or yard in the entire NCCLS study population.

Demographic data (child’s age, race/ethnicity, household income, parental educational level, type and age of residence) were collected through the questionnaire and statewide birth certificate files maintained by the California Department of Health Services (Sacramento, CA, USA) (Ma et al. 2002). For some analyses, annual household income was categorized as high (> $74,000), medium ($30,000–74,000), or low (< $30,000) according to California census classifications (U.S. Census Bureau 2012). A global positioning system was used to determine the latitude and longitude of the home, and a geographic information system was used to determine whether the residence was located in an urban, suburban, or rural area based on the 2000 U.S. census block characteristics (U.S. Census Bureau 2002; Ward et al. 2009).

U.S. EPA registration numbers collected from product labels were matched to the U.S. EPA Pesticide Product Information System (PPIS) databases that contain extensive data for every pesticide product licensed for sale in the United States. Active ingredients and their formulation, intended target pest, and potential toxicity were identified for each pesticide product inventoried (U.S. EPA 2011). We were not able to assess the concordance between self-reported and intended use of pesticides (as labeled by the manufacturer) because categories in the NCCLS questionnaire did not align perfectly with those of the U.S. EPA database (e.g., target pest, method of application).

We obtained information on chemical class by linking the databases of the U.S. EPA PPIS and the Pesticide Action Network (PAN; an organization that compiles information on pesticide class, toxicity, and health effects from official sources) (PAN 2011), using the U.S. EPA pesticide chemical codes (PC codes) that are unique to each active ingredient. The U.S. EPA PPIS database was managed in Microsoft Access (Microsoft Corporation, Redmond, WA, USA), the PAN database was managed in Microsoft Excel (Microsoft Corporation), and the final merged NCCLS/PPIS/PAN database was managed in SAS (version 9.2; SAS Institute Inc., Cary, NC, USA). The basic components of the data collection and databases as well as the detailed reformatting steps that were necessary for linking these databases are described in Supplemental Material [see Supplemental Material, Figure S1 and pp. 3–8 (http://dx.doi.org/10.1289/ehp.1204926)].

Information on the potential health effects of these chemicals was compiled from the websites of the U.S. EPA (U.S. EPA 2011), International Agency for Research on Cancer (IARC), PAN (PAN 2011), and National Pesticide Information Center (NPIC 2012).

*Statistical analysis.* Descriptive analysis of the household pesticides was performed using SAS. Univariate statistics for the number of pesticide products (median, interquartile range (IQR), range) were calculated for all of the 259 participating control households. The prevalence (presence or absence) of a particular pesticide characteristic (e.g., active ingredient, purpose of product use, storage area) was calculated on a per household basis among the 246 control households that stored at least one product. For example, if a household stored multiple insecticides that contained permethrin, it would contribute only once toward the prevalence of insecticides or permethrins.

Poisson regression models were used to identify statistically significant predictors of the number of products found in the households using STATA (version 10.0; StataCorp, College Station, TX, USA), and incidence rate ratios (IRR) were calculated. In this context, the IRR refers to the rate of pesticide products per household. We evaluated all of the demographic factors listed in Table 1, plus sex, first with a univariate analysis and then with a multivariate analysis. To determine improvement in the model fit, individual variables were dropped from the full model and likelihood ratio tests were conducted to compare the full model to the model without the variable being evaluated. We tested the fit of the final multivariate model by reintroducing the dropped variables individually and evaluating their contribution to model fit with a likelihood ratio test; none of the dropped variables were retained. A variable was retained as a significant predictor in the multivariate model if the *p*-value associated with the likelihood ratio test statistic was < 0.05. The final multivariate model was adjusted for ethnicity, household income, father’s or mother’s education, type and year of construction of residence, and time between the reference date and interview.

**Table 1:** Number of pesticide products by sociodemographic and household characteristics and Poisson regression model estimates of associations between each characteristic and the number of pesticides per home among 259 control households that completed an inventory, NCCLS (2001–2006).^*a*^

Characteristic	Households n (%)	No. of pesticide products per household		Poisson regression models		p-Valuec
				Univariate IRR (95% CI)	Multivariateb IRR (95% CI)	
		Median (IQR)	Range			
Overall	259 (100)	4 (2–7)	0–21
Child’s age at interviewd				1.03 (1.00, 1.06)		0.06
0–1 years	44 (17)	4 (2–7.5)	0–15
2–5 years	178 (69)	4 (2–7)	0–21
6–8 years	37 (14)	5 (3–9)	1–18
Child’s race/ethnicitye						< 0.0001
Non-Hispanic white	124 (48)	5 (3–9)	0–21	1.00 (reference)	1.00 (reference)
Hispanic	87 (34)	3 (1–5)	0–18	0.66 (0.58, 0.76)	0.83 (0.71, 0.96)
Non-Hispanic other	48 (19)	3.5 (1–6)	0–12	0.59 (0.50, 0.69)	0.61 (0.51, 0.72)
Annual household incomef						< 0.0001
> $75,000	125 (48)	5 (3–9)	0–21	1.00 (reference)	1.00 (reference)
$60–$74,000	29 (11)	5 (3–8)	0–21	0.90 (0.76, 1.07)	0.88 (0.74, 1.05)
$45–$59,000	35 (14)	4 (2–6)	1–19	0.89 (0.75, 1.05)	1.04 (0.86, 1.25)
$30–$44,000	28 (11)	2 (1–4)	0–15	0.59 (0.47, 0.73)	0.70 (0.55, 0.90)
$15–$29,000	28 (11)	2 (1–5)	0–9	0.54 (0.41, 0.70)	0.66 (0.49, 0.91)
< $15,000	14 (5)	2 (1–4)	0–4	0.33 (0.20, 0.53)	0.43 (0.26, 0.73)
Father’s educationf						0.02
Bachelor’s degree or higher	96 (37)	5 (3–8.5)	0–21	1.00 (reference)	1.00 (reference)
Some college or similar	75 (29)	5 (2–8)	0–21	0.96 (0.85, 1.09)	1.07 (0.93, 1.24)
High school or similar	61 (24)	3 (1–5)	0–21	0.61 (0.52, 0.72)	0.72 (0.60, 0.88)
None or elementary school	21 (8)	2 (1–3)	0–9	0.55 (0.41, 0.75)	0.97 (0.66, 1.40)
Mother’s educationf						0.04
Bachelor’s degree or higher	109 (42)	5 (3–9)	0–21	1.00 (reference)	1.00 (reference)
Some college or similar	78 (30)	4 (2–7)	0–16	0.76 (0.67, 0.86)	0.88 (0.76, 1.03)
High school or similar	58 (22)	3 (2–5)	0–18	0.66 (0.57, 0.78)	0.92 (0.75, 1.13)
None or elementary school	14 (5)	2 (1–3)	0–4	0.27 (0.14, 0.52)	0.44 (0.22, 0.87)
Residence typee						< 0.0001
Single-family residence	227 (88)	5 (2–8)	0–21	1.00 (reference)	1.00 (reference)
Duplex/townhouse	15 (6)	1 (1–3)	0–5	0.34 (0.22, 0.53)	0.44 (0.28, 0.69)
Apartment/condominium	12 (5)	1 (1–2)	0–9	0.41 (0.23, 0.72)	0.59 (0.33, 1.06)
Mobile home	4 (2)	1.5 (1–5)	1–5	0.62 (0.34, 1.13)	0.75 (0.41, 1.38)
Year residence builtf						0.65
1990–present	71 (27)	4 (2–8)	0–21	1.00 (reference)	1.00 (reference)
1985–1989	18 (7)	5 (2–7)	0–21	1.18 (0.94, 1.47)	1.12 (0.89, 1.42)
1980–1984	15 (6)	3 (2–8)	0–12	0.86 (0.66, 1.11)	0.80 (0.60, 1.04)
1970–1979	36 (14)	6 (4–9)	0–18	1.31 (1.11, 1.54)	1.46 (1.22, 1.74)
1960–1969	21 (8)	5 (3–7)	0–10	0.97 (0.78, 1.20)	0.91 (0.73, 1.14)
1940–1949	32 (12)	5 (3–10.5)	0–19	1.30 (1.10, 1.54)	1.18 (0.99, 1.41)
1939 or earlier	22 (8)	4.5 (2–8)	1–12	1.05 (0.85, 1.30)	1.00 (0.81, 1.25)
Unknown	16 (6)	4 (2–8)	1–10	0.71 (0.54, 0.93)	0.67 (0.50, 0.89)
Neighborhood typef						0.14
Urban	192 (74)	4 (2–8)	0–21	1.00 (reference)
Rural	38 (14)	4 (2–8)	0–13	0.92 (0.78, 1.08)
Suburban	27 (10)	2 (1–5)	0–12	0.60 (0.47, 0.75)
Time from reference date to interviewf						0.003
< 1 year	30 (12)	4.5 (2–8)	0–18	1.00 (reference)	1.00 (reference)
1–2 years	150 (58)	5 (2–8)	0–21	1.10 (0.92, 1.31)	1.09 (0.91, 1.30)
2–3 years	63 (24)	4 (2–7)	1–16	0.93 (0.77, 1.14)	0.95 (0.77, 1.17)
> 3 years	16 (6)	3 (1.5–4)	0–18	0.63 (0.45, 0.88)	0.59 (0.42, 0.83)
aPercentages do not sum to 100% due to missing values. bVariables included in the multivariate model were mutually adjusted for ethnicity, household income, father’s or mother’s education, type and year of construction of residence, and time between the reference date and interview. cp-Values are reported from the final multivariate model when effects were adjusted for other factors: from the trend test (for ordinal and continuous variables) or from the likelihood ratio test (for nominal variables). dModeled as a continuous variable. eModeled as a nominal variable. fModeled as an ordinal variable.									

*p*-Values are presented from the Wald trend test for ordinal variables (e.g., income, education, year of construction of residence, time between the reference date and interview) and continuous variables (e.g., age) and from the likelihood ratio test for nominal variables (e.g., ethnicity, type of residence). *p*-Values are reported from the final multivariate model when effects were adjusted for other factors. Age was modeled as a simple continuous variable in a univariate analysis and the *p*-value for the Wald test of the beta-coefficient was reported. The trend test for ordinal variables was performed by modeling as a continuous variable with a consecutive integer scores assigned to each category.

## Results

Table 1 describes the characteristics of the 259 control families who completed a household pesticide inventory. Ninety-five percent of participating households (246 of 259) stored at least one pesticide product (median, 4; IQR = 2–7). The median number of products differed significantly by race/ethnicity, household income, parental education, and housing type. The greatest number of products was found in non-Hispanic white households, households with higher income, higher educational level (for either the mother or father), and single family homes.

These findings were supported by multivariable Poisson regression models that demonstrated that household income, ethnicity, parental education, type and year of construction of residence, and time between the reference date and interview were significant independent predictors of the number of products found in the home. Child’s age at time of interview was not a significant predictor in the regression model, and we found no evidence of a systematic increase or decrease in the rate of pesticide storage with increasing age (*p*-value from test for trend = 0.06). Although type of neighborhood was not a significant predictor (*p*-value from likelihood ratio test > 0.05), suburban households (median, two products) stored about 40% fewer pesticide products and rural households (median, four products) stored 8% fewer products compared to urban households (median, four products). However there was insufficient evidence to conclude that rural, suburban, or urban residences were associated with a greater rate of pesticide storage (*p* = 0.14).

Compared with non-Hispanic white households, Hispanic households stored 17% fewer products (IRR = 0.83; 95% CI: 0.71, 0.96), whereas non-Hispanic (other than white) households stored nearly 40% fewer products (IRR = 0.61; 95% CI: 0.51, 0.72). Household income was a strong predictor of the number of products stored in our sample. Compared with families with the highest annual income (> $74,000), those with the lowest annual income (< $15,000) stored nearly 60% fewer products (IRR = 0.43; 95% CI: 0.26, 0.73).

Self-reported characteristics of pesticide use among 246 control households with products in storage are reported overall and by level of household income (Table 2). In general, the patterns of pesticide storage and use were similar between medium and high-income households compared with low-income households. The garage was the most common site of pesticide storage in medium- (42%) and high-income (45%) households, whereas the kitchen was the most common storage site among low-income families (46%). The lawn or garden was the most common site for pesticide use (28% overall), with little difference by income level. Using pesticide products indoors in the kitchen, bathroom, or family/living room was more frequent in the low-income than in the medium- and high-income families. Overall, ants were the most targeted pests (33%), followed by weeds (20%) and fleas (12%). Whereas products to kill weeds were used more frequently in medium- and high-income households, use of products targeting against ants, flies, rats, and indoor plant pests was more common in low-income households. Most families stored ready-to-use applications.

**Table 2:** Self-reported characteristics of residential pesticide use, overall and stratified by income,^*a*^ among 246 control households that stored at least one pesticide product, the NCCLS (2001–2006).^*b*^

Characteristic	Annual household income (%)			
	Low (n = 39)	Medium (n = 86)	High (n = 121)	All (n = 246)
Storage location
Garage	18	42	45	39
Kitchen	46	19	13	20
Detached shed	18	16	12	15
Utility Room	3	3	11	7
Bathroom	5	3	2	3
Closets	3	2	2	2
Basement	3	0	2	2
Vehicle	0	2	1	1
Barn	0	0	1	0
Other	3	10	11	9
Location of use (≤ 12 months)
Lawn/garden	33	29	26	28
Kitchen	23	17	17	18
Bathroom	21	10	11	12
Family room/living room/den	10	9	4	7
Bedroom or nursery	5	7	4	5
Foundation/soil	3	5	5	4
Dining room	3	6	3	4
Detached structures	3	2	1	2
Other outside	8	22	21	19
Other inside	5	6	7	7
Purpose of use
Ants	38	35	30	33
Weeds	10	26	20	20
Fleas	13	12	12	12
Flies	10	8	7	8
Slugs	0	6	7	6
Outdoor plants	5	2	5	4
Indoor plants	10	1	4	4
Rats	8	3	2	3
Bees	3	1	4	3
Termites	0	1	1	1
Other	8	8	13	11
Application method
Ready to use	56	37	43	43
Pour or spread granules	10	15	14	14
Compressed air sprayer	8	9	11	10
Shampoo, dip, apply	10	6	7	7
Bait-box	10	5	7	7
Hose-end sprayer	3	5	4	4
Dust, shake, blow	3	6	2	4
Applicator with handle	0	3	5	4
Bomb/fogger	0	7	1	3
Direct pour	0	1	2	2
Hand-held applicator	0	0	1	0.4
Slow release product	0	0	1	0.4
Flea or tick collar	0	0	1	0.4
Fly strip	0	0	0	0
Other	3	6	2	4
Time of last use
< 1 month ago	18	12	8	11
1 month to 1 year ago	64	48	52	52
> 1 year ago	13	19	26	22
Never used	5	15	9	11
Don’t know	0	7	4	4
Time period of use
Preconception (3 months before)	15	28	33	28
During pregnancy	15	37	44	37
1st trimester	13	26	33	27
2nd trimester	13	20	27	22
3rd trimester	13	28	30	26
Postpartum	28	51	55	50
0–1 years	21	42	47	41
1–2 years	21	43	50	43
2–3 years	21	50	49	45
aAnnual household income: low = < $30,000; medium = $30,000–$74,000; high = > $74,000. bPercentages do not sum to 100% because multiple products were used per household.				

Roughly half (52%) of the control households last used an inventoried pesticide product between 1 month and 1 year before the interview, whereas fewer families had used the products either < 1 month (11%) or > 1 year (22%) before the interview. Eleven percent of the households stored products that had never been used. Half of the households reported using pesticides after the child’s birth (50%), 37% during pregnancy, and 28% in the 3 months preceding conception (Table 2).

Of the 13 control households that did not store pesticides, most were Hispanic (*n* = 7) and lived in an urban area (*n* = 9) or a single family home (*n* = 9). There was no pattern in the number of products stored according to parental education or income, although only three households reported an annual income < $30,000 (data not shown).

Record linkage to public databases allowed us to ascertain information on active ingredients, chemical class, and health effects. Supplemental Material, Table S1 (http://dx.doi.org/10.1289/ehp.1204926), identifies the most common active ingredients overall, with their carcinogenicity classification, regardless of the pesticide type. Supplemental Material, Table S2, shows the most common chemical classes overall. Supplemental Material, Table S3, displays information on the targeted pest, the most common active ingredients (including synergists) and formulation types for each pest, and the locations of storage and use.

Table 3 lists the frequency of the most prevalent active ingredients identified in the inventoried products found in control households, classified by the most common types of pesticide (e.g., insecticide) and their common chemical classes (e.g., pyrethroid). Nearly half of the 246 households with products in storage possessed insecticides (46%) or miticides (44%). The most common chemical classes among the insecticides and miticides were pyrethroid (37%), organophosphorus (24%), and botanical insecticides (21%) (see Supplemental Material, Table S2 (http://dx.doi.org/10.1289/ehp.1204926), for definitions of the chemical classes according to the PAN), and the most common active ingredients were pyrethrin and permethrin insecticides (19% and 14% of households, respectively). Diazinon and chlorpyrifos, although removed from the market for residential use, were the most common organophosphorus insecticides inventoried (12% and 8%, respectively) (Table 3) and were also found in 20% and 12% of the households overall (see Supplemental Material, Table S1) in many different types of pesticides (see Supplemental Material, Table S3). A quarter of the households stored herbicides (24%); phosphonoglycine was the most common chemical class (14%) and glyphosate, isopropylamine salt (14%) was the most common active ingredient in this class (Table 3). Fungicides and other types of pesticides were found in ≤ 15% homes.

**Table 3:** Prevalence of common active ingredients, classified by type of pesticide and chemical class, inventoried in the 246 control households that stored at least one pesticide product, NCCLS (2001–2006).^*a*^

Pesticide type^*b*^	Chemical class^*c*^	Common active ingredients^*b,c*^
Insecticide (46%)/ miticide (44%)d	Pyrethroid (37%)	Permethrin (14%); d-trans allethrin (12%); imiprothrin (9%); Cypermethrin, beta (9%); tralomethrin (9%); tetramethrin (7%)
	Organophosphorus (24%)	Diazinon (12%); chlorpyrifos (8%); acephate (8%); disulfoton (3%); malathion (2%); phosmet (1%)
	Unclassified (23%)	Piperonyl butoxide (20%); triforine (7%); pyriproxyfen (2%)
	Botanical (21%)	Pyrethrins (19%); rotenone (1%); neem oil (1%)
	Dicarboximide (10%)	N-octyl bicycloheptene dicarboximide (10%)
Herbicide terrestrial (24%)	Phosphonoglycine (14%)	Glyphosate, isopropylamine salt (14%)
	Chlorophenoxy acid or ester (13%)	2,4-D, dimethylamine salt (11%); 2,4-dichlorophenoxyacetic acid (6%); MCPP, dimethylamine salt (5%)
	Benzoic acid (9%)	Dicamba, dimethylamine salt (8%); dicamba (2%)
	2,6-Dinitroaniline (4%)	Pendimethalin (2%); trifluralin (1%); oryzalin (1%)
	Aryloxyphenoxy propionic acid (2%)	Fluazifop-p-butyl (2%)
Fungicide (15%)	Unclassified (8%)	Triforine (8%)
	Organophosphorus (7%)	Acephate (7%)
	Pyrethroid (6%)	Resmethrin (5%); permethrin (1%)
	Inorganic copper (2%)	Copper sulfate (basic) (2%); copper ammonium complex (1%)
	Substituted benzene (2%)	Chlorothalonil (2%)
	Organotin, heavy metal (2%)	Fenbutatin-oxide (2%)
Molluscicide and tadpole shrimp (13%)	Aldehyde (9%)	Metaldehyde (9%)
	Inorganic (3%)	Iron phosphate (3%)
	N-Methyl carbamate (3%)	Carbaryl (3%)
Repellent or feeding depressant (11%)	Unclassified (10%)	DEET (9%); piperonyl butoxide (2%); dipropyl isocinchomeronate (1%)
	Botanical (2%)	Pyrethrins (2%); p-menthane-3,8-diol (0.4%)
	Dicarboximide (2%)	N-octyl bicycloheptene dicarboximide (2%)
	Pyrethroid (2%)	Permethrin (1%); d-trans allethrin (0.4%); phenothrin (0.4%)
Rodenticide (0.4%)	1,3-Indandione (0.4%)	Diphacinone (0.4%)
MCPP, methylchlorophenoxypropionic acid or Mecoprop. aThis list is not comprehensive because it contains only the most common pesticide types, chemical classes, and active ingredients. bAs listed in the U.S. EPA Pesticide Product Information System database. cAs listed in the PAN Pesticide database; detailed information on active ingredients can be found using the this database (PAN 2011). dInsecticides and miticides contained the same active ingredients and chemical classes and were therefore combined.		

## Discussion

Residential pesticides include a variety of active ingredients with differing toxicities and potential health effects. Few studies have inventoried pesticides in and around the home (Adgate et al. 2000; Bass et al. 2001; Whitmore et al. 1992). Therefore, the development of better methods to estimate exposure in population-based studies, including methods to obtain information on specific active ingredients, is needed. We reported findings from the control households enrolled in the NCCLS, which is unique among epidemiological studies for its integrated assessment of residential pesticide exposure; it included a comprehensive inventory of residential pesticides stored at time of interview with record linkage to publicly available databases, collection of home dust samples for analysis of pesticide residues, and questionnaire data. This approach provided detailed characteristics of the pesticides, including information on active ingredients and their chemical class, which can be used to inform self-reports about pest treatments.

At least one pesticide product (median, four) was found in 95% of the households (246 of 259), and roughly half (52%) used the products at least once within the year preceding the inventory. These findings are consistent with previous inventories; an average of 3.8 + 0.5 products (95% CI, 3.34–4.34) were inventoried among the 2,447 households included in the U.S. EPA National Home and Garden Pesticide Use Survey (Whitmore et al. 1992). All 107 households with children in a survey of a nonagricultural community in Arizona reported using stored pesticide products during the 6 months preceding the inventory (Bass et al. 2001), and most (93%) of the 308 households with children that stored products reported using them in the year preceding the inventory in a Minnesota study (Adgate et al. 2000).

Pest burden and consequently the numbers and types of pesticides used may be affected by temperature, season, geographic location, type of residence, and sociodemographic characteristics; the pest burden in our study area may differ from those of other areas with different climates. Overall, one-third of households in our survey used products to control ants, and one-fifth used products to control weeds and this pattern differed by sociodemographic characteristics.

Although poor housing conditions in low-income neighborhoods increase the chances of pest infestation and consequent pesticide usage (HUD 2006), this has not been consistently demonstrated in previous pesticide surveys in the homes of young children. Sociodemographic factors (i.e., race/ethnicity, income) did not predict pesticide storage and use patterns in a Minnesota study of predominantly non-Hispanic white households (Adgate et al. 2000), whereas an Arizona study (Bass et al. 2001) reported that their population of mainly low-income, Hispanic households used comparatively fewer products than households in other surveys (Adgate et al. 2000; Whitmore et al. 1992). In the NCCLS, lower household income and various sociodemographic variables (e.g., lower parental education, nonwhite ethnicity, and not living in single-family home) independently predicted a smaller number of stored products. Our ability to detect statistically significant differences by sociodemographic characteristics may be attributable partly to the larger variation of ethnic/racial and socioeconomic background in our study population in California compared with surveys conducted in other regions.

Although low-income households in our study used fewer products overall, they reported use of products against ants, flies, rats, and indoor plant pests more frequently than medium- and high-income households, which more frequently reported herbicide use. As predicted, using pesticide products to control pest infestations indoors (in the kitchen, bathroom, or family/living room) was more frequent in the low-income compared with the medium- and high-income families. Products were usually stored in the garage among medium- and high-income households (42–45%), whereas the kitchen was the most common storage site among low-income families (46%). Our findings are in agreement with an inventory study of mainly low-income, Hispanic households in Arizona that reported the kitchen as the most common site of pesticide storage (45%) (Bass et al. 2001). Low-income households in our survey had a higher opportunity for exposure to residential insecticides because they typically reported greater use of products indoors, which would potentially increase children’s exposures, compared with higher-income households that reported greater use of outdoor herbicides.

The types of products and the active ingredients present in our survey, identified via linkage to public databases, were similar to those found in previous inventories (Adgate et al. 2000; Bass et al. 2001). Half of the households had insecticides in storage (46%) and nearly one-fourth stored herbicides (24%). The most common chemical classes were pyrethroids (37%), organophosphorus (24%), and botanicals (21%). Pyrethrins, which are botanical insecticides made from crude extracts of plants from the chrysanthemum family, were the most common active ingredient in our survey (19%). Pyrethrins are potent insecticides, but are less persistent than pyrethroids (synthetic insecticides that are structurally derived from pyrethrins), which may result in lower exposure over time. The U.S. EPA has classified pyrethrins as suggestive carcinogens and permethrin—the most common pyrethroid insecticide in our sample—as a likely carcinogen (U.S. EPA 2011); these chemicals may also cause allergic reactions, asthma symptoms and neurotoxic effects and share a common mechanism of toxicity because of their common chemical structure (NPIC 2012). Chemicals banned for residential use were also found in the household pesticide products that we inventoried: Diazinon (banned in 2004) and chlorpyrifos (banned in 2001) were common active ingredients in insecticide products (PAN 2011).

Insecticides containing pyrethroid chemicals and pyrethrin are typically formulated with synergists, which were commonly found in the inventoried products. The synergists piperonyl butoxide and *N*-octyl bicycloheptene dicarboximide (commonly known as MGK 264), classified as possible carcinogens by the U.S. EPA (2011), were present in 42% and 22% of households, respectively. Inhalation of piperonyl butoxide can cause respiratory irritation and accumulation of fluid in the lungs (NPIC 2012). Although synergists do not have inherent pesticidal activity, they promote or enhance the effectiveness of certain active ingredients when combined (Bernard and Philogene 1993): piperonyl butoxide and *N*-octyl bicycloheptene dicarboximide inhibit the ability of insects and humans to detoxify pesticides (NPIC 2012).

Many of the other common active ingredients identified were classified as “suggested,” “possible,” or “likely” carcinogens by the U.S. EPA (see Supplemental Material, Table S1 (http://dx.doi.org/10.1289/ehp.1204926)] and may also cause adverse neurotoxic, developmental, and respiratory effects (NPIC 2012; PAN 2011). Although only active ingredients are listed in the U.S. EPA database (U.S. EPA 2011), so-called “inert”/“other” ingredients (chemicals that do not have direct activity against the target pest) often constitute a large percentage of a pesticide product. The term “inert”/”other” does not imply a lack of toxicity. Petroleum derivatives, which were a class of chemicals in pesticides found in 5% of the control households, are used as solvents for insecticides and often contain carcinogenic chemicals (U.S. EPA 2011). “Inert”/”other” ingredients may be carcinogenic, act synergistically with other components of the mixture, and have significant toxicological properties. Recent *in vitro* studies have shown that the herbicide glyphosate (Richard et al. 2005) and the pyrethroid bifenthrin (Hoffman et al. 2006) are less toxic to human cells in the placenta and immune system, respectively, than commercially equivalent products that occur in a mixed formulation, suggesting that differences in the “inert”/”other” ingredients may explain the differential effects. Information on “inert”/”other” ingredients present in pesticide formulations is generally considered to be proprietary by the manufacturer and not readily available to the public, thereby limiting the full evaluation of these pesticides.

A strength of the NCCLS household inventory is the high participation among the population-based controls. It is plausible that many of the products contributing to pesticide exposure were actually inventoried since this inventory was conducted among residentially stable households with young children. Restricting our analysis to this subset of our sample may limit the ability to generalize to California as a whole or to other populations, but nonetheless provides useful information on residential pesticides. Although this inventory likely missed some products that were used in the past and not replaced (such as bombs or foggers) and products that are typically not stored (such as flea collars), this information was captured via self-reports of pest treatments and will be used in future analyses.

The NCCLS is one of just a few studies to inventory residential pesticides (Adgate et al. 2000; Bass et al. 2001; Whitmore et al. 1992), interview participants about pesticide use, and collect dust samples in households with young children. Combining the information from self-report and the inventory about the product type, method of application, location of storage and use, and timing of use, with the active ingredients and chemical classes identified from the PPIS and PAN databases and household dust samples, will allow for a more comprehensive assessment of the role of residential exposure to pesticides in the risk of childhood leukemia in future analyses and inform approaches to modeling risk of a wide range of health outcomes in young children (Zartarian et al. 2000).

## Conclusions

We found that multiple pesticide products were commonly used and stored in a sample of California households with young children and that the number of pesticide products stored increased with income level. By linking the pesticide products found to available databases, we also determined the active ingredients to which children in our study were likely to have been exposed. It is notable that many of the commercial products inventoried contain active ingredients that may have carcinogenic, respiratory, neurotoxic, and developmental effects. The data presented here strengthen current knowledge on non-occupational pesticide exposure in the general population, particularly in children, and will inform the development of models for risk assessment and future analyses of childhood leukemia.

## Supplemental Material

(86 KB) PDF
