# Supplementary material for: Characterization of Residential Pesticide Use and Chemical Formulations through Self-Report and Household Inventory: The Northern California Childhood Leukemia Study
Source: Environ Health Perspect. 2012 Oct 24;121(2):276–82. doi: 10.1289/ehp.1204926 (PMC3569677; doi:10.1289/ehp.1204926)
Supplement: (86 KB) PDF [file ehp.1204926.s001.pdf]

Supplemental Material

Characterization of Residential Pesticide Use and Chemical Formulations through Self-Report and Household Inventory: The Northern California Childhood Leukemia Study

Neela Guha[1,2], Mary H. Ward[3], Robert Gunier[1], Joanne S. Colt[3], C. Suzanne Lea[4], Patricia Buffler[1] and Catherine Metayer[1]

Authors affiliations:
[1]School of Public Health, University of California at Berkeley, Berkeley, California 94704
[2]International Agency for Research on Cancer, Lyon, France 69008
[3]Division of Cancer Epidemiology and Genetics, National Cancer Institute, Bethesda, Maryland 20892
[4]Department of Public Health, Brody School of Medicine, East Carolina University, Greenville, North Carolina 27834

Table of Contents

Supplemental Material, Figure S1. Overview of the data collection and record linkage for the household pesticide inventory in the NCCLS (2001-2006).	Page 2
Supplemental Material. Process of matching United States Environmental Protection Agency (USEPA) registration numbers to the USEPA Pesticide Product Information System (PPIS) databases.	Page 3
Supplemental Material, Table S1. Prevalence (%) of the most common active ingredients identified in inventoried products among 246 control households that stored at least one pesticide product, the NCCLS (2001-2006).	Page 9
Supplemental Material, Table S2. Most common chemical classes identified in inventoried products among 246 control households that stored at least one pesticide product enrolled in the NCCLS (2001-2006).	Page 10
Supplemental Material, Table S3. Prevalence (%) of pesticide products classified by target pest, active ingredients, formulation, and areas of storage and use identified in the 246 households that stored at least one pesticide product in the NCCLS (2001-2006)	Page 12
Supplemental Material, References	Page 14

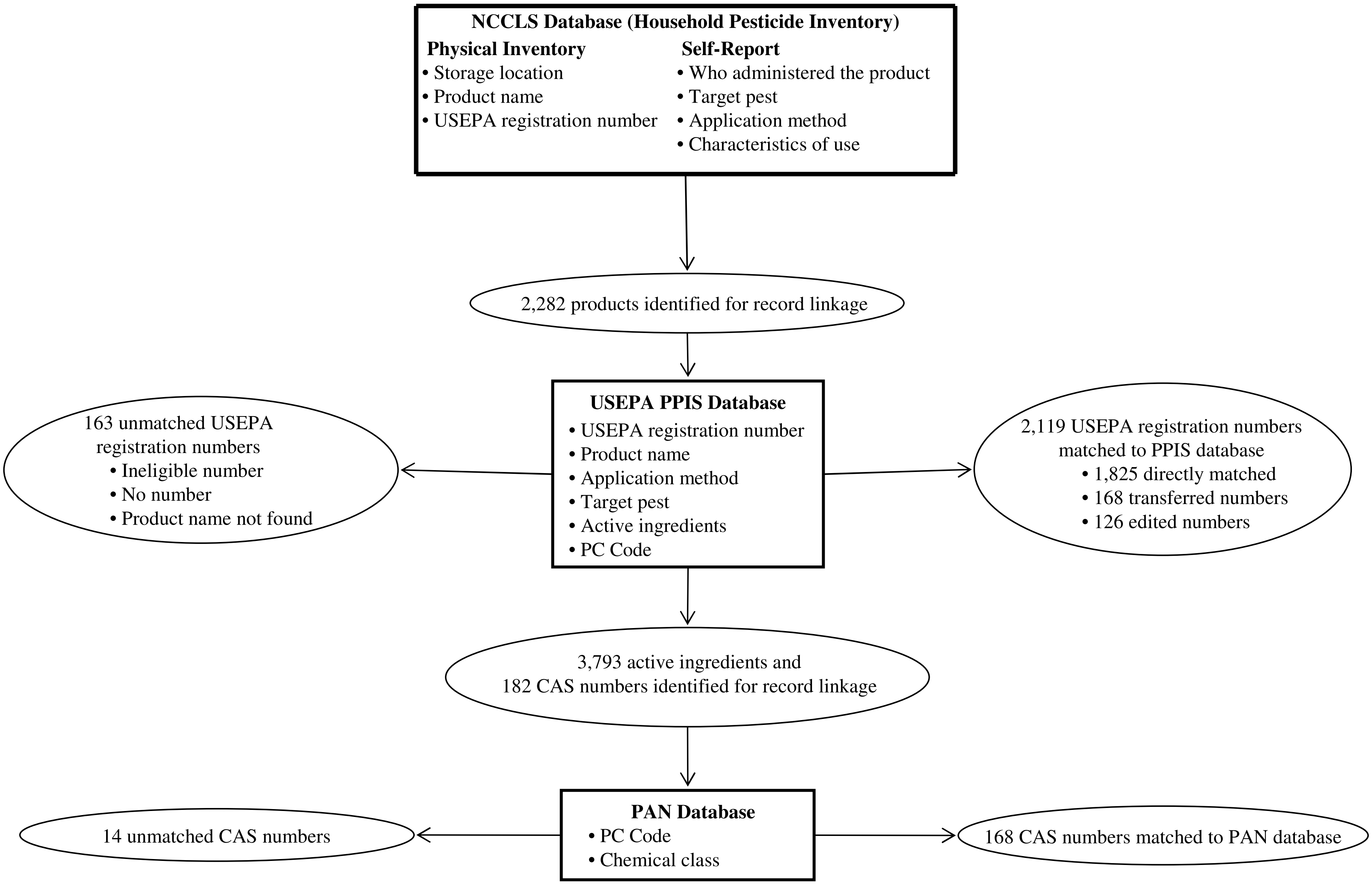

Supplemental Material, Figure S1. Overview of the data collection and record linkage for the household pesticide inventory in the NCCLS (2001-2006).

Supplemental Material. Process of matching United States Environmental Protection Agency (USEPA) registration numbers to the USEPA Pesticide Product Information System (PPIS) databases.

More than 2000 pesticide products (n= 2,282) were inventoried in total and 93% (n= 2, 119) were matched to the USEPA PPIS database: 1825 (86%) of the USEPA registration numbers were directly matched, 168 (8%) were found to have transferred USEPA registration numbers (products that were assigned a new USEPA registration number due to transfer from one firm to another) and 126 (6%) were matched using an edited USEPA registration number (due to expired pesticide registrations and data entry errors). We were not able to link 163 products (7%) to the USEPA PPIS database mainly because of an illegible USEPA registration number, no reported USEPA registration number, and/or the product name was not found.

One hundred and eighty-two CAS numbers were obtained from the active ingredients identified in the USEPA PPIS database and the majority (n=168) were matched to a chemical class listed in the Pesticide Action Network (PAN) database. We describe below the steps that were undertaken to match the USEPA registration numbers, collected from pesticide product labels, to the USEPA PPIS databases.

A. Understanding the data: Description of the USEPA registration number

USEPA registration numbers are up to 19 numerical digits in length and are composed of a basic registrant's firm number, a product number, an optional distributor's firm number, and an optional California revision code, all separated by a hyphen (e.g. 4822-10-ZB for Off! Insect Repellent). The basic registrant's firm number (up to 7 digits in length) identifies the company that is the primary registrant with the USEPA. California assigns their own unique firm numbers to companies that register products which are not required to be registered by the USEPA. The

product number (up to 5 digits in length) is generally assigned sequentially to each company's individual product as it is registered with the USEPA.

The distributor's number (up to 7 digits in length) identifies any company that is marketing a product owned by another company (generally the primary registrant). For a product registered in California, this number represents the company that holds the license for pesticide registration within the state.

The California revision code is a sequence of two alphabetic letters that may or may not appear on the actual product label. This code results from the state of California's requirement for companies to register and license individual brand names: a single product may have many brand names registered within the state and thus the revision code creates a unique identifier for each product. Unique revision codes are assigned to each product to allow for identification of the specific brand name in question. The California Department of Pesticide Regulations (CDPR) (http://www.cdpr.ca.gov/docs/label/epadef.htm) and USEPA (http://www.epa.gov/oppfead1/labeling/lrm/chap-14.htm#II) list further details of the components of an USEPA registration number.

B. Steps for data management and linkage of the Northern California Childhood Leukemia Study (NCCLS) and USEPA PPIS databases

1. Format the USEPA PPIS database: USEPA registration numbers are stored in the "Reg_Nr" field of the USEPA PPIS "products" table as a numeric string, concatenated without hyphens; it is comprised of the firm number and product number and consists of 19 numerical digits (or 12 digits numbers without the distributor's number). Because the "Reg_Nr" field did not appear initially to conform to the documented USEPA standard format (See preceding

section on *Description of the USEPA registration number*), we made several assumptions to infer the USEPA registration number.

The USEPA registration number has, at bare minimum, a single-digit "firm ID" number and a single-digit product number. For USEPA registration numbers smaller than 12 (or 19) digits, we assumed that the registration numbers in the USEPA PPIS database conformed to the standard numbering format (See section on *Description of the USEPA registration number*). Given that there were no registration numbers ("Reg_Nr") in the USEPA PPIS database with fewer than six digits, with many of the registration numbers of the form #0000# (with the # representing digits from 1-9), it appeared that the USEPA concatenated the seven digit firm number and the five digit product number and dropped any leading zeros from this number.

2. NCCLS data editing: All of the USEPA registration numbers recorded from product labels during the home interview were verified by comparing the USEPA registration numbers to the product names using the websites of the CDPR http://www.cdpr.ca.gov/docs/label/labelque.htm#regprods, USEPA http://oaspub.epa.gov/pestlabl/ppls.home, and the Pesticide Action Network (PAN) http://www.pesticideinfo.org/Search_Products.jsp.

3. Format the NCCLS database: The following steps were taken to format the USEPA registration numbers in the NCCLS database in order to link to the USEPA PPIS database:

a. Parse the USEPA registration number into individual components: We identified the individual components of the reported USEPA registration numbers and parsed the fields into firm number, product number, distributor number, and California revision codes in order to reformat for linking the two databases.

b. Remove California revision codes: We assumed that we could strip the two-letter revision codes from the USEPA registration numbers reported in our database without loss of specificity, since these are unique identifiers (required only by the state of California) used to distinguish specific brands. The USEPA database does not make brand-level distinctions. Thus we assumed that removing the revision code from the reported registration numbers would not adversely influence the percentage of USEPA registration numbers that matched between those recorded during the household inventory and those existing in the USEPA PPIS.

c. Remove distributor number: The USEPA registration number was retained as recorded in our database if it contained only two components (assumed to be the firm number and the product number). If the reported USEPA registration number contained three components (as outlined above), then we retained only the first two components (assumed to be the firm number and the product number). We omitted the third component from all USEPA registration numbers recorded in our database since we assumed that it represented the optional distributor number that is irrelevant for linking to the USEPA PPIS database.

d. Further reformatting: We concatenated the firm number and product number to conform to the "Reg_Nr" format. The USEPA registration numbers recorded from the home product inventory did not contain leading zeros whereas the USEPA PPIS database used leading zeros, where necessary, to differentiate the product number from firm number in the consolidated "Reg_Nr" field (see assumptions above). Consequently, we concatenated a string of four zeros to the beginning of the product number and retained the right-most five characters from the resulting string to represent the product number according to the USEPA PPIS convention. We then concatenated the product number to the end of the firm

number to create the composite USEPA registration number in order to link to the USEPA PPIS database.

4. Linking the USEPA registration numbers in the NCCLS and USEPA PPIS databases: Once the USEPA registration numbers in the NCCLS database had been edited and reformatted, they could then be linked to the USEPA PPIS databases to retrieve information on active ingredients (information on unlisted inert ingredients may be retrieved from Material Safety Data Sheets), toxicity, intended use, and other useful information and links. A description on the available USEPA PPIS datasets, data dictionaries, and other useful information can be found at: http://www.epa.gov/opppmsd1/PPISdata/index.html. Information to import the USEPA datasets into MS Access databases can be found at: http://www.bmckay.com/ppisatut.htm.

Only 7% (n = 163) of the 2,282 records were unlinked and therefore we evaluated four possible reasons: expired pesticide registrations, erroneously recording the CAS (Chemical Abstract Service) registration number instead of the USEPA registration number from the product labels, data entry errors, and transferred products.

a. Expired pesticide registrations: The datasets that are available for download from the USEPA PPIS database contain only active pesticide registrations. However, the version of the USEPA PPIS database that is available only on the website contains USEPA registration numbers that are both active and inactive. Therefore, we evaluated unmatched USEPA registration numbers against the expired or cancelled registrations that are still accessible via the online version of the USEPA PPIS database.

b. Erroneous misidentification of the CAS registration number: Many pesticide products may have the CAS registration number printed on the labels in addition to the USEPA

registration number. Thus it is possible that unmatched USEPA registration numbers reported were in fact the CAS registration number rather than the USEPA registration number. We undertook this analysis however we were not able to match additional unlinked USEPA registration numbers to the CAS registration numbers listed in the USEPA PPIS database.

c. Data entry errors: We conducted a visual scan of unmatched records to determine if there were any likely data entry errors that could account for mismatches. For example, some USEPA registration numbers may have been entered without hyphens and thus we parsed the USEPA registration numbers to get the firm number and the product number to allow for reformatting. Typing errors incurred during the data entry process were also corrected.

d. Transferred products: USEPA registration numbers may change when a firm/registrant transfers a product to another firm/registrant. We searched for unlinked USEPA registration numbers in a table of transferred products found in the USEPA PPIS database. We were able to determine the current USEPA registration number where we found matches. We then replaced the unmatched USEPA registration number with the current USEPA registration number (found in the transferred products table) and subsequently re-ran our match query to further link products between the two databases.

Supplemental Material, Table S1. Prevalence (%) of the most common active ingredients identified in inventoried products[a,b] among 246 control households that stored at least one pesticide product, the NCCLS (2001-2006).

Active Ingredient	% Homes	Use Type	Chemical Class	USEPA Carcinogen Classification[c]	IARC Carcinogen Classification[d]
Pyrethrins	43	Insecticide	Botanical	Suggestive	Not listed
Piperonyl butoxide	42	Synergist	Unclassified	C, Possible	3, Unclassifiable
Glyphosate, isopropylamine salt	39	Herbicide	Phosphonoglycine	Not listed	Not listed
Permethrin	35	Insecticide	Pyrethroid	Suggestive	3, Unclassifiable
N-octyl bicycloheptene dicarboximide	22	Synergist	Dicarboximide	C, Possible	Not listed
Cypermethrin, beta	22	Insecticide	Pyrethroid	C, Possible	Not listed
D-trans Allethrin	21	Insecticide	Pyrethroid	Suggestive	Not listed
2,4-D, dimethylamine salt	21	Herbicide	Chlorophenoxy acid or ester	D, Unclassifiable, ambiguous data	Not listed
C9-C11 alkyl, oligomeric D-glucopyranoside	20	N/A	N/A	Not listed	Not listed
Diazinon	20	Insecticide	Organophosphorus	Not Likely	Not listed
Metaldehyde	20	Molluscicide	Aldehyde	Suggestive	Not listed
Tetramethrin	18	Insecticide	Pyrethroid	C, Possible	Not listed
Tralomethrin	15	Insecticide	Pyrethroid	Not listed	Not listed
DEET	15	Insect Repellent	N,N-dialkylamide[e]	D, Unclassifiable, inadequate data	Not listed
Phenothrin	15	Insecticide	Pyrethroid	Not Likely	Not listed
Dicamba, dimethylamine salt	15	Herbicide	Benzoic acid	D, Unclassifiable, inadequate data	Not listed
Carbaryl	13	Insecticide, Plant Growth Regulator, Nematicide	N-Methyl Carbamate	Likely	3, Unclassifiable
Bifenthrin	12	Insecticide	Pyrethroid	C, Possible	Not listed
Chlorpyrifos	12	Insecticide, Nematicide	Organophosphorus	E, Unlikely	Not listed
2,4-D	11	Herbicide, Plant Growth Regulator	Chlorophenoxy acid or ester	D, Unclassifiable, ambiguous data	Not listed

[a]Using record linkage to the USEPA Pesticide Product Information System database.
[b]Detailed information on active ingredients can be found using the Pesticide Action Network Pesticide database: http://www.pesticideinfo.org/Search_Chemicals.jsp
[c] More information on the USEPA carcinogen classification can be found here : http://www.epa.gov/iris/search_human.htm
[d] More information on the IARC carcinogen classification can be found here: http://monographs.iarc.fr/ENG/Classification/index.php
[e] National Pesticide Information Center.

Supplemental Material, Table S2. Most common chemical classes identified in inventoried products among 246 control households that stored at least one pesticide product enrolled in the NCCLS (2001-2006)[a,b].

Chemical Class	n (%)[c]	Definition of Chemical class[a]
Pyrethroid	189 (77)	Synthetic insecticides (typically cyclopropane carboxylates) structurally similar to pyrethrins, which are naturally occurring insecticidal compounds. Many pyrethroids are suspected endocrine disruptors.
Unclassified[d]	140 (57)	PAN has evaluated the chemical structure and determined that it does not fit into any of the chemical classes listed here.
Botanical	124 (50)	Pesticides derived from plants. These pesticides are typically a plant's natural defense against insects or fungi. Examples are nicotine and pyrethrins. Some of these compounds are quite toxic.
Organophosphorus	103 (42)	Compounds, mostly insecticides, that contain a phosphorus atom bound to organic substituents, either alkyl or alkoxy groups. Most organo-phosphorus pesticides are cholinesterase inhibitors which cause neurotoxicity in both insects and humans.
Phosphonoglycine	96 (39)	Herbicidal organophosphorus compound. These compounds inhibit a plant-specific enzyme and have low acute toxicity to mammals. Glyphosate and its salts and esters (active ingredients in Roundup products) belong to this class of compounds.
Phenoxy herbicide	77 (31)	
Chlorophenoxy acid or ester	66 (27)	Herbicidal compounds such as 2,4-D and 2,4,5-T. Many chlorophenoxy compounds are suspected endocrine disruptors.
Aryloxyphenoxy propionic acid	11 (5)	Definition not provided.
Dicarboximide	54 (22)	Fungicidal compounds. Vinclozolin and iprodione are examples.
Carbamate (N-Methyl carbamate)	52 (21)	Compounds (mostly insecticides) with an N-methyl amide functional group. Most N-methyl carbamates are strong cholinesterase inhibitors which cause neurotoxicity in both insects and humans. N-methyl carbamates are the most acutely toxic of all carbamate pesticides.
Heavy metal	51 (21)	
Inorganic-arsenic	18 (7)	Arsenic-containing compounds that do not have arsenic-carbon bonds. These compounds are generally extremely toxic, causing acute toxicity, cancer, and developmental toxicity.
Organoarsenic	11 (5)	Arsenic-containing compounds with an organic moiety bound directly to arsenic. These compounds are generally extremely toxic, with many used as chemical warfare agents.
Inorganic-copper	10 (4)	Definition not provided.
Organotin	10 (4)	Tin-containing compounds with an organic moiety bound directly to tin. These compounds are generally extremely acutely toxic and are also known to be endocrine disruptors. Examples are tributyltin or triphenyltin salts.
Inorganic-zinc	2 (0.8)	Zinc-containing compounds that do not have zinc-carbon bonds. Most are used as microbiocides or fungicides.
Aldehyde	48 (20)	Definition not provided.
Inorganic	47 (19)	Any chemical compound not containing hydrocarbon moieties and not one of the toxic metals. Inorganic compounds of toxic metals--mercury, arsenic, cadmium, chromium, tin, lead, and silver--are listed separately (see below). Examples of inorganics are lime, phosphoric acid, and sulfuryl fluoride.
Benzoic acid	42 (17)	Compounds with a benzoic acid functional group. Many of these are herbicides. Examples are chloramben and dicamba.
Chloro-nicotinyl	23 (9)	Definition not provided.

Supplemental Material, Table S2 (cont.)

Chemical Class	n (%)[c]	Definition of Chemical class[a]
Soap	23 (9)	Compounds with surfactant or detergent properties. Used as insecticides and adjuvants.
Coumarin	21 (9)	Rodenticides that act as anticoagulants. Cinnamic acid lactone structure.
Bipyridylium	17 (7)	Herbicides containing two pyridine rings, joined through a C-C bond. Most are acutely toxic to mammals. Paraquat is a bipyridilium compound.
2,6-Dinitroaniline	17 (7)	Herbicidal compounds containing a dinitroaniline functional group. While these compounds are not acutely toxic to animals, many of them are possible human carcinogens.
Pyrazole	13 (5)	A relatively new class of insecticide that is persistent in the environment and may pose substantial ecological risks. Chlorfenapyr (Pirate) is an example.
Substituted benzene	12 (5)	Fungicidal compounds with a benzene ring substituted with various substituents. Some of these compounds are very toxic wood preservatives such as pentachloro-nitrobenzene (PCNB) and hexachlorobenzene.
Petroleum derivative	12 (5)	Compounds derived from crude oil through a distillation process. Frequently used as solvents, adjuvants, and insecticides. Depending on the level of refinement, petroleum derivatives often contain carcinogenic substances.

[a]As listed in the Pesticide Action Network database. Detailed information on active ingredients can be found using http://www.pesticideinfo.org/Search_Chemicals.jsp

[b]Percentages don't sum to 100% because each household may store pesticides of multiple chemical classes.

[c]Chemical classes present in at least 5% of control households.

[d]PAN has evaluated the chemical structure and determined that it does not fit into any of the chemical classes listed here.

Supplemental Material, Table S3. Prevalence (%) of pesticide products classified by target pest, active ingredients, formulation, and areas of storage and use identified in the 246 households that stored at least one pesticide product in the NCCLS (2001-2006)[a]

Target Pest[b]	Active Ingredients or Synergists[c]	Formulation Types[c]	Storage Area[b]	Area used[b]
Ants-Cockroaches (35%)	Imiprothrin (12%)<br>Cypermethrin, beta (11%)<br>D-trans Allethrin (9%)<br>Piperonyl butoxide (9%)<br>Tralomethrin (9%)	Pressurized Liquid (49%)<br>Ready-to-Use Solution (15%)<br>Emusifiable Concentrate (10%)<br>Granular (10%)<br>Dust (3%)	Kitchen (35%)<br>Garage (35%)<br>Utility Room (14%)<br>Other (6%)<br>Detached shed (5%)<br>Closets (2%)<br>Basement (1%)	Kitchen (21%)<br>Bathroom (14%)<br>Family/Living Room (11%)<br>Lawn/Garden (11%)<br>Bedroom/Nursery (7%)<br>Dining Room (6%)<br>Foundation/Soil (4%)<br>Detached Structures (2%)<br>Other Outside (14%)<br>Other Inside (10%)
Weeds (21%)	Glyphosate, isopropylamine salt (19%)<br>2,4-D, dimethylamine salt (13%)<br>Dicamba, dimethylamine salt (10%)<br>2,4-D (7%)<br>Mecoprop-P (6%)	Soluble Concentrate (30%)<br>Ready-to-Use Solution (26%)<br>Granular (18%)<br>Emulsifiable Concentrate (5%)<br>Pressurized Liquid (4%)	Garage (56%)<br>Detached Shed (29%)<br>Utility Room (4%)<br>Other (4%)<br>Kitchen (2%)<br>Closet (2%)<br>Basement (2%)	Lawn/Garden (67%)<br>Other Outside (30%)<br>Other Inside (1%)<br>Foundation/Soil (1%)
Slugs-Snails (11%)	Metaldehyde (51%)<br>Iron phosphate (17%)<br>Carbaryl (17%)<br>Diazinon (6%)<br>Neem oil (3%)	Granular (57%)<br>Pelleted/Tableted (30%)<br>Ready-to-Use Solution (7%)<br>Soluble Concentrate (3%)	Garage (53%)<br>Detached Shed (29%)<br>Other (15%)<br>Utility Room (4%)	Lawn/Garden (83%)<br>Other Outside (10%)<br>Other Inside (7%)
Flies-Mosquitoes (9%)	Piperonyl butoxide (18%)<br>DEET (18%)<br>Pyrethrins (12%)<br>Phenothrin (8%)<br>D-Allethrin (6%)	Pressurized Liquid (48%)<br>Ready-to-Use Solution (33%)<br>Emulsifiable Concentrate (7%)<br>Dust (4%)	Garage (32%)<br>Kitchen (21%)<br>Bathroom (18%)<br>Utility Room (11%)<br>Closets (7%)<br>Other (7%)<br>Vehicle (4%)	Lawn/Garden (19%)<br>Other Outside (19%)<br>Other Inside (13%)<br>Bathroom (11%)<br>Family/Living Room (11%)<br>Kitchen (11%)<br>Bedroom/Nursery (6%)<br>Detached Structure (6%)<br>Dining Room (4%)
Fleas-Ticks (9%)	Piperonyl butoxide (14%)<br>Pyrethrins (14%)<br>S-Methoprene (13%)<br>N-octyl bicycloheptene dicarboximide (11%)<br>Permethrin (11%)	Ready-to-Use Solution (56%)<br>Pressurized Liquid (15%)<br>Dust (11%)<br>Emulsifiable Concentrate (11%)<br>Impregnated Materials (4%)	Garage (40%)<br>Utility Room (24%)<br>Kitchen (12%)<br>Bathroom (8%)<br>Detached Shed (8%)<br>Closets (4%)<br>Other (4%)	Other Outside (29%)<br>Other Inside (18%)<br>Lawn/Garden (18%)<br>Family/Living Room (12%)<br>Bathroom (9%)<br>Kitchen (9%)<br>Bedroom/Nursery (3%)<br>Foundation/Soil (3%)

Supplemental Material, Table S3. (cont.)

**Target Pest**[b]	**Active Ingredients or Synergists**[c]	**Formulation Types**[c]	**Storage Area**[b]	**Area used**[b]
Outdoor Plant-Trees (6%)	Triforine (19%)<br>Disulfoton (13%)<br>Acephate (13%)<br>Chlorothalonil (10%)<br>Resmethrin (10%)	Soluble Concentrate (23%)<br>Emulsifiable Concentrate (18%)<br>Granular (18%)<br>Pressurized Liquid (14%)<br>Oils with no added pesticide (9%)	Garage (53%)<br>Detached Shed (40%)<br>Other (7%)	Lawn/Garden (90%)<br>Other Inside (13%)
Indoor Plant-Trees (6%)	Resmethrin (18%)<br>Acephate (18%)<br>Triforine 18%)<br>Diazinon (9%)<br>Piperonyl butoxide (9%)	Pressurized Liquid (29%)<br>Ready-to-Use Solution (24%)<br>Emulsifiable Concentrate (19%)<br>Granular (10%)<br>Pelleted/Tableted (5%)	Utility Room (27%)<br>Garage (27%)<br>Detached Shed (27%)<br>Kitchen (13%)<br>Other (7%)	Lawn/Garden (76%)<br>Other Outside (24%)
Bees-Wasps-Hornets (5%)	D-trans Allethrin (25%) Propoxur (13%)<br>Chlorpyrifos (13%)<br>Phenothrin (13%)<br>Tralomethrin (13%)	Pressurized Liquid (77%)<br>Ready-to-Use Solution (15%) Emulsifiable Concentrate (15%)<br>Dust (4%)	Garage (46%)<br>Utility Room (23%)<br>Kitchen (8%)<br>Basement (8%)<br>Detached Shed (8%)<br>Other (8%)	Other Oustide (27%)<br>Lawn/Garden (20%)<br>Other Inside (13%)<br>Detached Structure (10%)<br>Kitchen (10%)<br>Bedroom/Nursery (7%)<br>Family/Living Room (10%)<br>Bathroom (3%)<br>Dining Room (3%)
Rats-Mice-Gophers (4%)	Brodifacoum (54%)<br>Diphacinone (15%)<br>Carbon (8%)<br>Sodium nitrate (8%)<br>Sulfur (8%)	Granular (55%)<br>Pelleted/Tableted (36%)<br>Impregnated Materials (9%)	Garage (55%)<br>Kitchen (27%)<br>Detached Shed (18%)	Other Inside (47%)<br>Kitchen (20%)<br>Lawn/Garden (20%)<br>Dining Room (7%)<br>Other Outside (7%)
Termites-Ants (2%)	Sodium tetraborate (pentahydrate) (25%)<br>Diazinon (25%)<br>Mineral oil/petroleum distillates/ solvent refined light (25%)<br>Permethrin (25%)	Emulsifiable Concentrate (50%)<br>Granular (25%)<br>Ready-to-Use Solution (25%)	Garage (50%)<br>Kitchen (25%)<br>Other (25%)	Other Outside (40%)<br>Foundation/Soil (20%)<br>Kitchen (20%)<br>Lawn/Garden (20%)
Other (9%)	Methyl nonyl ketone (8%)<br>2,4-D (5%)<br>MCPP (5%)<br>Disulfoton (5%)<br>Diazinon (5%)	Granular (21%)<br>Ready-to-Use Solution (21%)<br>Emulsifiable Concentrate (14%)<br>Pressurized Liquid (10%)<br>Soluble Concentrate (10%)	Garage (61%)<br>Detached Shed (17%)<br>Kitchen (13%)<br>Other (9%)	Lawn/Garden (43%)<br>Other Outside (22%)<br>Bedroom/Nursery (8%)<br>Family/Living Room (8%)<br>Bathroom (5%)<br>Detached Structure (3%)<br>Dining Room (3%)<br>Foundation/Soil (3%)<br>Kitchen (3%)<br>Other Inside (3%)

[a]This table is not be comprehensive since the most common active ingredients, formulation types, and storage and use locations are listed
[b]As listed in the NCCLS questionnaire
[c]As listed in the USEPA Pesticide Product Information System database.

References

NPIC. 2012. National Pesticide Information Center Factsheets. http://npic.orst.edu/factsheets/. [accessed 27 April 2012].

PAN. 2011. Pesticide Action Network Pesticides Database - Chemicals. http://www.pesticideinfo.org/Search_Chemicals.jsp. [accessed 27 April 2012].

USEPA. 2011. Pesticide Product Information System. http://www.epa.gov/pesticides/PPISdata/. [accessed 27 April 2012].
